# Glutathione Metabolism, Mitochondria Activity, and Nitrosative Stress in Patients Treated for Mandible Fractures

**DOI:** 10.3390/jcm8010127

**Published:** 2019-01-21

**Authors:** Jan Borys, Mateusz Maciejczyk, Bożena Antonowicz, Adam Krętowski, Jarosław Sidun, Emilia Domel, Jan Ryszard Dąbrowski, Jerzy Robert Ładny, Katarzyna Morawska, Anna Zalewska

**Affiliations:** 1 Department of Maxillofacial and Plastic Surgery, Medical University of Bialystok, Sklodowskiej M.C. 24, 15-274 Bialystok, Poland; jjbb7@wp.pl (J.B.); emilia.domel@gmail.com (E.D.); 2 Department of Physiology, Medical University of Bialystok, Mickiewicza 2c Str., 15-233 Bialystok, Poland; 3 Department of Oral Surgery, Medical University of Bialystok, Sklodowskiej M.C. 24a Str., 15-276 Bialystok, Poland; antbo1@wp.pl or bozena.antonowicz@umb.edu.pl; 4 Clinical Research Centre, Medical University of Bialystok, Sklodowskiej M.C. 24a Str., 15-276 Bialystok, Poland; adamkretowski@wp.pl; 5 Department of Material and Biomedical Engineering, Faculty of Mechanical Engineering, Bialystok University of Technology, 15-351 Bialystok, Poland; j.sidun@pb.edu.pl (J.S.); j.dabrowski@pb.edu.pl (J.R.D.); 6 Department of Emergency Medicine and Disasters, Medical University of Bialystok, 37 Szpitalna Street, 15-767 Bialystok, Poland; ladnyjr@wp.pl; 7 Department of Conservative Dentistry, Medical University of Bialystok, Sklodowskiej M.C. 24a Str., 15-276 Bialystok, Poland; kmorawska1009@gmail.com (K.M.); azalewska426@gmail.com (A.Z.)

**Keywords:** titanium fixations, oxidative stress, nitrosative stress, metallosis

## Abstract

The aim of the study was to evaluate the effect of titanium bone fixations on mitochondrial activity, reactive oxygen species (ROS) production, glutathione metabolism, and selected markers of oxidative/nitrosative stress in the periosteum-like tissue of patients treated with mandible fractures. The study group consisted of 30 patients with bilateral fractures of the mandible body eligible for surgical treatment. Our study is the first one that indicates disturbances of mitochondrial activity as well as a higher production of ROS in the periosteum-like tissue covering titanium fixations of the mandible. We also found significantly higher levels of reduced glutathione and enhanced activity of glutathione reductase in the periosteum homogenates of patients in the study group compared to the control group. Levels of nitrosative (S-nitrosothiols, peroxynitrite, nitrotyrosine) and oxidative stress biomarkers (malondialdehyde, protein carbonyls, dityrosine, kynurenine, and N-formylkynurenine) were statistically elevated in periosteum-like tissue covering titanium fixations. Although exposure to titanium fixations induces local antioxidant mechanisms, patients suffer oxidative damage, and in the periosteum-like tissue the phenomenon of metallosis was observed. Titanium implants cause oxidative/nitrosative stress as well as disturbances in mitochondrial activity.

## 1. Introduction

In recent years, a surgical procedure utilizing titanium miniplates and screws has become the standard treatment offered in jaw fractures. With the implementation of osteosynthesis, the period needed for intermaxillary fixation—A practice that is quite inconvenient for the patient—May be shortened, and the function of the stomatognathic system restored more quickly [1]. Despite the high biocompatibility of titanium and its alloys, the necessity of removing titanium implants is being increasingly discussed due to the risk of remote side effects associated with the long-term settlement of biomaterials in the body [2,3]. According to the literature, in some patients with titanium implants the phenomenon of metallosis is observed [4]. Metallosis is defined as tissue deposition of metallic particles that are released from the implant due to friction and electrochemical corrosion [5,6]. Both the presence of the titanium implant and its wear products may be the cause of the body’s immune-inflammatory response to the metallic foreign body [7,8]. This may result in premature loss of the implant (i.e., fixations, prostheses of the joints), or entail the need for additional surgical procedures to maintain the implant in the body [7,8]. Moreover, the released metal particles may increase the production of free radicals and lead to redox imbalances favoring oxidation reactions [9,10]. This condition causes oxidative/nitrosative stress, in which cell components are damaged by an increased activity of reactive oxygen species (ROS) and reactive nitrogen species (RNS) [11]. It is believed that oxidation damage of cell membranes, their altered permeability and dysfunction of structural and enzymatic proteins may disturb the regeneration processes of the body [11,12].

In order to counteract the disturbances of redox homeostasis, living organisms have developed specialized antioxidant mechanisms protecting them against overproduction of ROS and RNS. One of the most important intracellular antioxidants is glutathione, which regulates the expression of numerous genes and participates in the metabolism of proteins, thus playing a significant role in regeneration and repair processes [13,14]. It is assumed that redox imbalances induced by titanium may affect regenerative processes during bone fracture healing [6,15]. Similarly, the pathomechanism of the development of inflammatory reactions around titanium fixations, observed even many years after osteosynthesis, is still unclear. Sources of ROS overproduction are also unknown in patients treated with titanium mandibular implants. Due to the lack of studies assessing the influence of titanium on the main antioxidant mechanism in the body (reduced glutathione/oxidized glutathione system) as well as the formation of ROS and RNS, the aim of our study was to evaluate the effect of titanium fixations on glutathione metabolism, mitochondrial activity, ROS production, and selected markers of nitrosative and oxidative stress in the periosteum-like tissue of patients treated with mandible fractures.

## 2. Materials and Methods

### 2.1. Patients

The experiment was conducted in accordance with the Declaration of Helsinki (1964). The protocol of the study was approved by the Bioethics Committee of the Medical University of Bialystok, Poland (R-I-002/3/2-16). All patients were informed about the manner of conducting the study and agreed in writing to participate in the experiment.

The study included 60 patients treated at the Department of Maxillofacial and Plastic Surgery of the Medical University of Bialystok, Poland. Patients with normal body weight (18.5 ≤ body mass index (BMI) ≤ 24.5), without systemic (type 1 and 2 diabetes, obesity, cardiovascular diseases, cancer), autoimmune, mental, and infectious diseases and without thyroid, lung, liver, kidney, or gastrointestinal disorders were screened for the study.

The study group consisted of 8 women and 22 men aged 20–28 (*n* = 30; mean age 23 years and 3 months) with bilateral fractures of the mandible body eligible for surgical treatment. The causes of fractures in the patients were: beatings (59.1%), sports (22.5%), traffic accidents (10.8%), accidents at work (4.1%), and unfortunate falls (3.5%). Patients with other types of mandibular fractures (singular fracture, fracture of mandibular ramus or condyle) were not included in the study. Upon admission to hospital, patients underwent radiological and biochemical examinations and had fragments of their mandible adjusted and immobilized by means of titanium plates and screws. Each patient was treated with two 4- or 6-hole plates and 16–24 screws (MEDGAL Sp. z o.o., Księżyno, Poland) made of Ti6Al4V titanium alloy containing 90% titanium, 6% aluminum, and 4% vanadium. Immediately after the procedure and up until 3–5 months after osteosynthesis, all the patients were fed a balanced diet established by a dietician (2000 kcal comprised of 55% carbohydrates, 30% fat, and 15% protein). Moreover, every 2 weeks the patients had check-up appointments with an experienced maxillofacial surgery specialist (Jan Borys). In the physical examination no symptoms of acute or chronic dermatitis or mucositis (such as reddening, edema, inflammatory infiltration, abscess, or purulent fistula) were observed in the vicinity of the implants. No enlargement of the surrounding lymph nodes or allergy symptoms such as edema, changes on the skin or oral mucosa were observed either. The reasons for removing the plates were: discomfort connected with a palpably felt fixation (4 patients), cold hypersensitivity (5 patients), or a planned implant treatment after tooth loss (12 patients). In the remaining patients, titanium fixations were removed in order to avoid any potential risk of allergic reactions to a foreign body in the future. In all individuals, implants were removed at the explicit request of the patient.

The control group consisted of 30 generally healthy subjects selected on the basis of age and gender (8 women and 22 men; mean age 23 years and 7 months), who were operated on due to complete retention of lower wisdom teeth. Tooth eruption was not complicated by inflammation, and the teeth were removed only upon orthodontic indications. The impacted mandibular third molars were categorized as being of the mesioangular (20), vertical (7), or horizontal (3) position, class III C according to the Pell and Gregory [16,17] classifications. A fragment of periosteum was collected in the vicinity of oblique line of the mandible, after incising and dissecting the mucoperiosteal flap, before exposing and removing the impacted mandibular third molars.

The criteria for excluding patients from the control and study group were: inflammatory complications and disorders of the union of the fractured bone, wounds of soft tissues, injuries of the skull, chest, abdomen or extremities, as well as previous operations necessitated by bone fractures. Smokers, alcoholics, patients with oral diseases (gingivitis, periodontitis, or active odontogenic infection foci), and people taking antibiotics, glucocorticosteroids, non-steroidal anti-inflammatory drugs, iron preparations, vitamins, and dietary supplements were also excluded from the experiment.

### 2.2. Periosteum-Like Tissue Collection

In the study group, the patients had their titanium fixations removed 3–5 months after osteosynthesis. The procedure was performed under local anesthesia of 2% lignocaine with epinephrine (Polfa, Warsaw, Poland) by a specialist experienced in maxillofacial surgery (Jan Borys). The research material (periosteum-like tissue) consisted of small fragments (2 × 8 mm, 0.5 mm thick) of gray-pigmented tissue adhering to the titanium implants excised as a standard procedure during the removal of the mandibular bone fixations. In the control group, healthy periosteum collected during exposure and extraction of retained third lower molars was used for the study. The collected tissues were immediately immersed in liquid nitrogen and then stored at −80 °C until the performance of assays (but not longer than 6 months).

### 2.3. Homogenates Preparation

On the day of the assays, the tissues were slowly thawed at +4 °C, weighed, and fragmented with surgical scissors. A portion of the tissue was placed in ice-cold phosphate-buffered saline (PBS, 9 mL per 1 g tissue), and the remaining was placed in mitochondria isolation buffer. In order to protect the samples from oxidation and proteolysis, butylated hydroxytoluene and protease inhibitor (1 tablet/10 mL PBS; Complete Mini, Roche, France) were added to the homogenates. The tissues were homogenized with a blade-type homogenizer (Omni TH, Omni International, Kennesaw, GA, USA) at a speed of 5000 rpm (the beaker was stored in an ice bath). The obtained tissue suspensions were sonified in an ice bath (1800 J/sample, 3 × 20 s; sonifier UP 400S, Hielscher, Teltow, Germany) and then centrifuged for 10 minutes at a speed of 5000× *g* at 4 °C. The supernatant fluid was preserved for further studies. In order to determine the concentration of total glutathione, oxidized (GSSG) and reduced (GSH), tissues were homogenized on ice in 5% solution of 5-sulfosalicylic acid (1:10, *v*/*v*) [18].

### 2.4. Mitochondria Isolation

To isolate mitochondria, tissues were homogenized in ice-cold mitochondria isolation buffer (250 mM sucrose, 5 mM Tris-HCl, and 2 mM EGTA (ethylene glycol bis (2-aminoethyl) tetraacetic acid; pH 7.4; 9 ml buffer per 1 g of tissue). Homogenates were centrifuged (500× *g*, 10 min, 4 °C) and the resulting supernatants were centrifuged twice at 8000× *g* (10 min, 4 °C). The mitochondria pellet was resuspended in the isolation medium and immediately analyzed.

### 2.5. Redox Assays

The performed analyses included: determination of mitochondrial function (complex I activity, complex II activity, cytochrome c oxidase (COX), citrate synthase (CS)) and free radical production (mitochondrial hydrogen peroxide formation, 2,7-dichlorodihydrofluorescein diacetate (DCFH-DA) ROS production assay); determination of antioxidant capacity (total glutathione, reduced glutathione (GSH), oxidized glutathione (GSSG), GSH/GSSG ratio, glutathione reductase (GR)); determination of nitrosative stress (S-nitrosothiols, peroxynitrite, and nitrotyrosine); determination of oxidative damage to lipids (malonylodialdehyde (MDA)) and proteins (protein carbonyls (PC)); and determination of protein glyco-oxidative products (dityrosine, kynurenine, N-formylkynurenine, and tryptophan). All redox biomarkers were measured in the homogenates of mandibular periosteum. The activity of mitochondrial complexes was determined in isolated mitochondria (see Section 2.6).

All assays were performed in duplicate samples and standardized to mg of total protein. The absorbance/fluorescence was measured using Infinite M200 PRO Multimode Microplate Reader, Tecan (Tecan Group Ltd., Männedorf, Switzerland). The concentration of total protein was measured by the bicinchoninic acid (BCA) method. The commercial kit Thermo Scientific PIERCE BCA Protein Assay (Rockford, IL, USA) was used in accordance with the manufacturer’s instructions. 

### 2.6. Mitochondrial Activity and ROS Production

The activity of complex I (E.C. 1.6.5.3) was measured colorimetrically based on 2,6-dichloroin-dophenol reduction by electrons accepted from decylubiquinol [19]. The activity of complex II (E.C. 1.3.5.1) was assayed according to Rustin et al. [20] by measuring the activity of succinate-ubiquinone reductase. The activity of cytochrome c oxidase (E.C. 1.9.3.1; COX) was determined at 550 nm by measuring the oxidation of reduced cytochrome c [21]. The activity of citrate synthase (E.C. 2.3.3.1; CS) was estimated colorimetrically in the reaction with 5-thio-2-nitrobenzoic acid, which is generated from 5,5′-dithiobis-2-nitrobenzoic acid during synthesis of citrate synthase [22]. The production of hydrogen peroxide (H_2_O_2_) was assayed fluorimetrically by measuring the increase in fluorescence at 530/590 nm due to the reaction of Amplex Red with H_2_O_2_ [23]. The rate of H_2_O_2_ production was calculated using a standard curve of H_2_O_2_ stabilized solution. The rate of ROS production was measured fluorimetrically using 2,7-dichlorodihydrofluorescein diacetate (DCFH-DA) [24]. DCFH-DA is de-esterified to 2,7-dichlorodihydrofluorescein (DCFH) by oxygen radicals and ROS production rate was calculated from the calibration curve for DCFH. The activity of complex I and II, CS, COX, and hydrogen peroxide production was determined in isolated mitochondria. DCFH-DA assay was performed in periosteum homogenates immediately after sample collection [25].

### 2.7. Glutathione Metabolism

To assess the antioxidant capacity, we focused on the glutathione system. The concentration of total glutathione was determined by colorimetric method using enzymatic reaction with reduced nicotinamide adenine dinucleotide phosphate (NADPH), 5,5’-dithiobis-(2-nitrobenzoic acid) (DTNB), and glutathione reductase (GR) [18]. The increase in absorbance was measured at a 412-nm wavelength. The total glutathione concentration was calculated from the calibration curve prepared for the reduced glutathione (GSH) solutions. The concentration of oxidized glutathione (GSSG) was determined by a colorimetric method similar to the one used for the total glutathione [18] assay, the difference being that prior to the determination the samples had been thawed and neutralized to pH 6–7 with 1 M chlorhydroltriethanolamine (TEA) and then incubated with 2-vinylpyridine (to inhibit glutathione oxidation). The concentration of oxidized glutathione was calculated from a calibration curve prepared for GSSG solutions. The reduced glutathione (GSH) concentration was calculated from the difference between the concentrations of total glutathione and oxidized glutathione: GSH = total glutathione − GSSG. Oxidation/reduction potential was calculated based on the ratio of reduced glutathione to oxidized glutathione (GSH/GSSG ratio). The concentration of total thiols was assayed colorimetrically by the Ellman method, in which 5,5’-dithiobis-(2-nitrobenzoic acid) (DTNB) was reduced to 2-nitro-5-thiobenzoic acid with participation of thiol groups [26]. The absorbance of the samples was determined at 412 nm wavelength. The activity of glutathione reductase (GR, E.C. 1.8.1.7) was determined colorimetrically by measuring the decrease in NADPH absorbance at 340 nm [27]. One unit of GR activity was defined as the quantity of enzyme which catalyzes the oxidation of 1 µmol NADPH per 1 min.

### 2.8. Nitrosative Stress

The concentration of S-nitrosothiols was measured using the colorimetric method described by Wink et al. [28], based on the reaction of the Griess reagent with Hg^2+^ mercury ions. The absorbance of the resulting complex was measured at 490 nm. The concentration of peroxynitrite was assayed according to the method described by Beckman et al. [29]. The basis of the assay was peroxynitrite-mediated nitration of phenol resulting in nitrophenol formation. The absorbance of the obtained complex was measured at a 320-nm wavelength. The concentration of nitrotyrosine was determined by the ELISA method using the commercial kit Nitrotyrosine ELISA Immundiagnostik AG (Bensheim, Germany) according to the manufacturer’s instructions included in the package. 

### 2.9. Oxidative Damage Products

The concentration of malondialdehyde (MDA) was measured spectrophotometrically using thiobarbituric acid (TBA) [30]. 1,1,3,3-tetraethoxypropane was used as standard. The absorbance of supernatants was measured at a 535-nm wavelength. The concentration of carbonyl groups (PC) in oxidatively modified proteins was assayed by the colorimetric method based on the reaction with 2,4-dinitrophenylhydrazine (2,4-DNPH) [31]. The absorbance of the samples was measured at 360 nm wavelength. PC content was calculated using an absorption coefficient for 2,4-DNPH = 22,000 M^−1^ cm^−1^. To detect protein glyco-oxidative products (dityrosine, kynurenine, N-formylkynurenine, and tryptophan), samples were diluted in 0.1 M H_2_SO_4_ (1:10, *v*/*v*). Fluorescence at 330/415 nm (dityrosine), 365/480 nm (kynurenine), 325/434 nm (N-formylkynurenine), and 95/340 nm (tryptophan) was measured, and the results were normalized to fluorescence of 0.1 mg/mL quinine sulfate in 0.1 M H_2_SO_4_ [15].

### 2.10. Energy Dispersive X-Ray Spectroscopy (EDS)

To determine metallosis, the research material consisting of fragments of gray pigmented periosteum-like tissue (2 × 2 mm) was taken from the former fracture site at the titanium miniplates in the projection of the first screw. The samples were tested for existing chemical makeup with the use of the Hitachi S-3000N electron scanning microscope, equipped with NSS X-ray spectrometer (Noran System Six) and a freezing table for biological specimens. The EDS detector recorded the composition of elements in the area of electron beam penetration at three points: in the center and in two points diagonally, approximately 0.5 mm from the center of the periosteal fragment. The tests were conducted at the speeding voltage of 25 kV and measuring time of 100 s. The energy values of the characteristic radiation allow for elemental identification in the sample, while the intensity (peak heights) allows for quantitative analysis. We determined the percentage content of carbon (C), oxygen (O), sodium (Na), silicon (Si), phosphorus (P), sulfur (S), chlorine (Cl), aluminum (Al), titanium (Ti), and vanadium (V) in the analyzed tissues.

### 2.11. Statistical Analysis

Statistical analysis was performed using the GraphPad Prism (GraphPad Software, La Jolla, CA, USA) and Statistica 10.0 system (StatSoft, Cracow, Poland). The D’Agostino-Pearson test and the Shapiro–Wilk test confirmed the normal distribution of results and therefore parametric tests were used for further analyses. A Student’s *t*-test was used to compare the results between the study group and the control group. In cases of abnormal distribution, the Whitney–Mann U test was applied. The associations between various parameters were tested by the Pearson’s correlation coefficient. Statistical significance was established at *p* ≤ 0.05. Data were expressed as mean ± standard deviation (SD). The number of patients was set based on a previously conducted pilot study (the power of the test was set at 0.9).

## 3. Results

### 3.1. Mitochondria Activity and ROS Production

**Figure 1:**
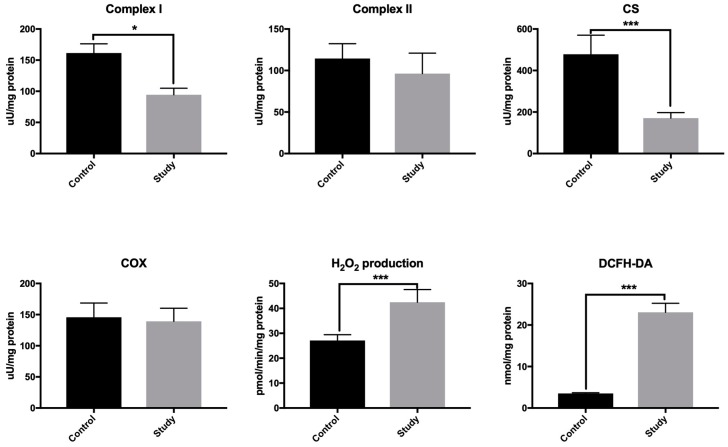
Mitochondria activity and reactive oxygen species (ROS) production in the study group periosteum-like tissue and the control group periosteum. COX: cytochrome c oxidase; CS: citrate synthase; CDFH-DA: 2,7-dichlorodihydrofluorescein diacetate; H_2_O_2_: hydrogen peroxide. A Student’s *t*-test was used to compare the results between the study group and the control group. * *p* < 0.05, and *** *p* < 0.0005.

In order to assess mitochondria function, we evaluated the activity of respiratory complexes and the key mitochondrial enzymes (i.e., cytochrome C oxidase (COX) and citrate synthase (CS)). The activity of mitochondrial complex I (–77.8%) and CS (–166.7%) was significantly reduced in the periosteum-like tissue of patients from the study group compared to the control group periosteum (Figure 1). However, the activity of complex II and COX did not statistically differ between the patients from the study group and the control. Hydrogen peroxide production (+55.6%) as well as rate of ROS formation (DCFH-DA assay) (+633.3%) was statistically elevated in the periosteum-like tissue covering titanium fixations as compared to the control group periosteum (Figure 1). This indicates an increased production of ROS not only in the periosteum-like tissue but also mitochondria of patients in the study group. Therefore, the source of ROS in these patients may be disturbances in respiratory complex I. It is well known that the decrease in complex I activity results in overproduction of free radicals [32].

### 3.2. Glutathione Metabolism

**Figure 2:**
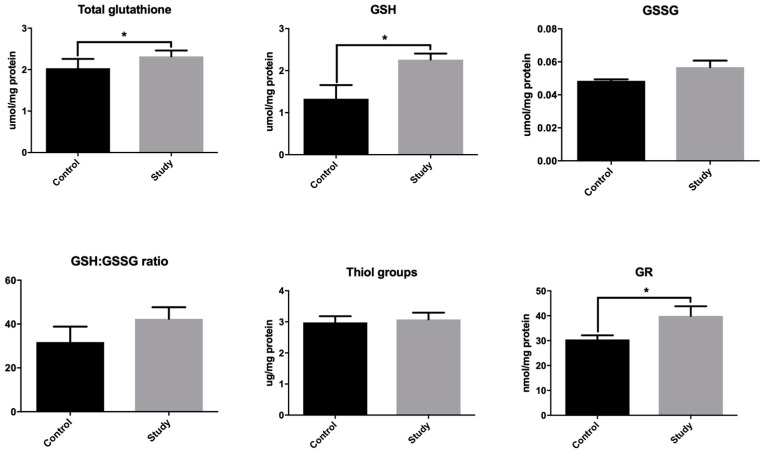
Glutathione metabolism in the study group periosteum-like tissue and the control group periosteum. GR: glutathione reductase; GSH: reduced glutathione; GSSG: oxidized glutathione; GSH/GSSG ratio: reduced glutathione/oxidized glutathione ratio. A Student’s *t*-test was used to compare the results between the study group and the control group. * *p* < 0.05.

One of the most important cellular antioxidants is the glutathione system, which includes the reduced (GSH) and oxidized (GSSG) forms of glutathione, as well as glutathione reductase (GR), which converts GSSG to GSH. In our study, we found a significantly higher concentration of total glutathione (+102%) in the periosteum-like tissue covering the titanium constructs compared to the control group periosteum (Figure 2). GSSG concentration did not differ significantly between the study and the control group. A statistically higher concentration of GSH (+70%) was found in the mandibular periosteum-like tissue of the study group, while the redox potential calculated from the ratio of reduced glutathione concentration to oxidized glutathione concentration (GSH/GSSG ratio) did not differ significantly between the two groups (Figure 2). Similarly, the concentration of total thiols (marker of oxidative protein damage) did not change significantly between the study and the control group. For patients with titanium implants, GR activity in the periosteum-like tissue was significantly higher compared to the controls (Figure 2). The increase in mandibular antioxidative systems (↑GSH, ↑GR) suggests an adaptive response to the increased production of ROS in patients treated with titanium miniplates and screws. No significant differences in the GSH/GSSG ratio and concentration of thiol groups indicates moderate changes in the redox balance.

### 3.3. Nitrosative Stress

**Figure 3:**
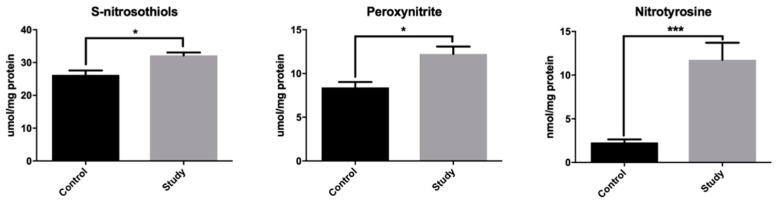
Nitrosative stress in the study group periosteum-like tissue and the control group periosteum. A Student’s *t*-test was used to compare the results between the study group and the control group. * *p* < 0.05, and *** *p* < 0.0005.

To assess the nitrosative stress, we used S-nitrosothiols (nitric oxide (NO) donors), peroxynitrite (oxidant formed from NO and superoxide anion (O_2_^−^)), as well as nitrotyrosine (marker of nitrosative damage to proteins). The concentration of S-nitrosothiols (+17.9%), peroxynitrite (+45%), and nitrotyrosine (+411%) was significantly higher in the mandibular periosteum-like tissue around titanium fixations compared to the control group periosteum (Figure 3). Thus, we are the first to confirm the induction of nitrosative stress in patients treated with titanium mandibular implants.

### 3.4. Oxidative Modification Products

**Figure 4:**
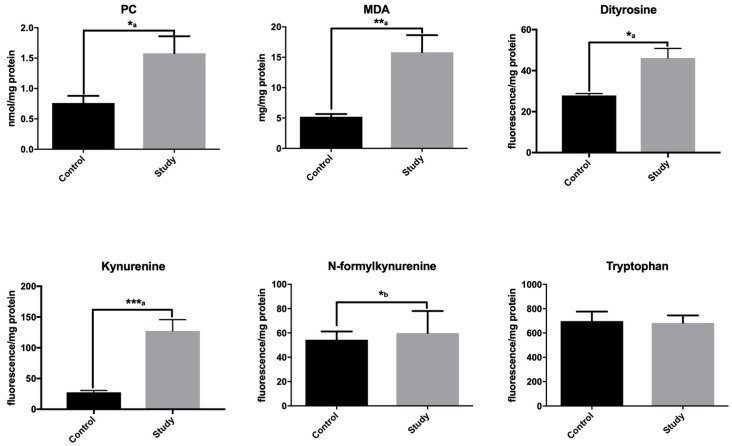
Oxidative damage in the study group periosteum-like tissue and the control group periosteum. MDA: malondialdehyde; PC: protein carbonyls. ^a^ Student’s *t*-test; ^b^ Mann–Whitney U test. * *p* < 0.05, ** *p* < 0.005, and *** *p* < 0.0005.

In patients with mandibular fractures, a significantly higher concentration of MDA (lipid oxidation product) (+204%) was found around titanium fixations compared to the control group. MDA is also a marker of osteoclastic activity [33], which may suggest intensified osteolytic processes around titanium miniplates and screws. The concentration of protein oxidative damage marker (protein carbonyls, PC) was significantly higher in periosteum-like tissue (+108%) of the study group than in the control group periosteum (Figure 4). The content of protein glyco-oxidative products (dityrosine, kynurenine, and N-formylkynurenine) was also notably higher in the mandible periosteum-like tissue of patients treated with titanium implants compared to the controls (Figure 4). Therefore, our results indicate not only oxidative damage to lipids and proteins, but also increased glyco-oxidation processes.

### 3.5. Energy-Dispersive X-Ray Spectroscopy (EDS)

**Figure 5:**
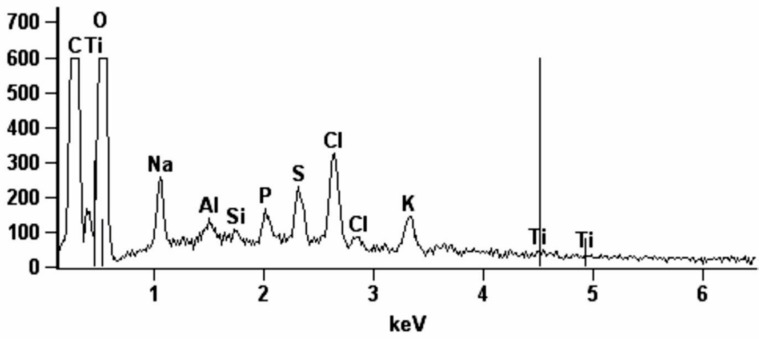
Exemplary energy-dispersive X-ray spectroscopy (EDS) spectrum of elements in the periosteum-like tissue of the study group.

Table 1 and Figure 5 show the percentage content of the elements determined in the periosteum-like tissue of patients treated with Ti4Al4V mandibular fixations and the periosteum of healthy controls. Particularly noteworthy is the significantly higher content of Ti, Al, and V in the periosteum-like tissue of patients from the study group compared to the periosteum of control patients in which these elements were not found. Thus, the results of our research confirm metallosis in the periosteum-like tissue of patients treated with titanium miniplates and screws.

### 3.6. Correlations

In patients with titanium mandibular fractures, a positive correlation was found between the ROS production rate (DFHA-DA assay) and GSH concentration (*r* = 0.78, *p* = 0.001), as well as between peroxynitrite and nitrotyrosine levels (*r* = 0.71, *p* = 0.001). These may indicate that increased antioxidant defense (↑GSH) in the periosteum-like tissue of patients from the study group is an adaptative reaction to the higher formation of ROS (↑DCHA-DA). However, an increase in GSH levels may not effectively protect against nitrosative damage (↑peroxynitrite, ↑nitrotyrosine) in these patients.

## 4. Discussion

In our previous studies, we have demonstrated cellular redox imbalance and increased oxidative damage in the periosteum as well as plasma/erythrocytes of patients treated with titanium miniplates and screws [6,15]. However, the presented research is the first study to indicate disturbances of mitochondrial activity and higher production of ROS in the periosteum-like tissue covering titanium fixations of the mandible. Additionally, we have shown alternations in glutathione metabolism as well as enhanced nitrosative stress.

Recognition of the physicochemical properties of titanium led to a significant progress in many areas of biomedicine, due to its favorable biomechanical properties, osteointegratability, corrosion resistance, and biocompatibility with tissues and body fluids [1]. However, no metal or its alloy is completely indifferent to the human body. In our study, microscopic analysis of mandible fixations in patients treated with titanium miniplates and screws reveals signs of mechanical corrosion in the removed implants. Notably, all patients in the study group had intraoperative grey staining of the periosteum-like tissue adjacent to the removed miniplates and screws. According to Torgersen et al. [34], the concentration of metallic particles in the tissue surrounding the implant in the range of 100–300 ppm is the cause of its visible staining.

Not only mechanical factors but also electrochemical corrosion may lead to the release of metal ions from the surface of implants. This is facilitated by reactions with body fluids and is largely dependent on the pH of the environment in which the implant is located. In the course of anode corrosion, molecular oxygen is additionally reduced, which leads to intensive formation of oxygen free radicals [6,35], also demonstrated by the results of our study (↑DCFH-DA). Additionally, raised glutathione levels and increased activity of GR in the mandible of patients treated with fixations made of Ti4Al4V titanium alloy may indicate a local adaptive response to the overproduction of ROS and RNS. Particularly, this is confirmed by the positive correlation between ROS production rate (DCFH-DA assay) and GSH concentration in the mandibular periosteum-like tissue. It is well known that GSH plays an important role in the prevention of metal toxicity [36,37]. Reduced glutathione demonstrates the ability to chelate ions or particles of heavy metals once they penetrate the cell. 

Despite increased antioxidant protection (↑GSH, ↑GR), exposure to Ti6Al4V alloy leads to oxidation of proteins (↑PC, ↑tryptophan degradation products, ↑nitrotyrosine) and lipids (↑MDA) caused by oxidative and nitrosative stress. Thus, intensified production of glutathione does not effectively protect against free radical modifications of the periosteum-like tissue at the site of titanium plate implantation.

The main cell component that is oxidized is protein [38]. In our experiment, we demonstrated increased contents of dityrosine, kynurenine, and N-formylkynurenine—Products of oxidation of aromatic tyrosine and tryptophan—In the periosteum-like tissue of the study group patients. This may result in abnormal structure of the organic matrix of a bone and thus weaken its biomechanical properties. Indeed, it is well known that tryptophan degradation products may inhibit differentiation of osteoprogenitor cells into osteoblasts and have a negative effect on bone fracture healing [15,39]. Additionally, of all reactive oxygen and nitrogen species, peroxynitrite (ONOO^−^) plays a particularly important role in oxidative protein damage [40,41]. ONOO^−^ not only oxidizes aromatic amino acid residues, but also leads to the formation of protein carbonyl groups [41]. This seems to be confirmed by the presented results (↑nitrotyrosine, ↑PC), particularly by the positive correlation between the levels of peroxynitrite and nitrotyrosine in the mandible periosteum-like tissue in contact with Ti6Al4V titanium alloy. Moreover, it was shown that increased ONOO^−^ levels in peri-implant tissues are associated with the inflammatory response of macrophages to wear debris, which may lead to bone resorption and loss of the implant [42]. Our study is the first to indicate increased levels of nitrosative stress biomarkers (S-nitrosothiols, peroxynitrite, and nitrotyrosine) in the periosteum-like tissue of patients treated with Ti4Al4V titanium alloy. It is suggested that increased production of peroxynitrite may disturb metabolism and bone tissue remodeling in the vicinity of titanium fixations. In an in vitro study, it has been demonstrated that the activation of macrophages by titanium wear debris is accompanied by stimulation of inducible nitric oxide synthase (iNOS), which causes the release of large amounts of NO with other inflammatory mediators (interleukin-1 (IL-1), IL-6, tumor necrosis factor (TNF)-α) [43]. This leads to bone resorption in osteoclast cell cultures [43].

Additionally, our study indicates that ROS production in patients treated with titanium implants may also be caused by alternations in mitochondrial activity (↓complex I, ↓CS). In disturbed mitochondria, the reduction of oxidized NADP decreases, which impairs the glutathione system and increases production of ROS and RNS [32]. In present study, this was confirmed by the increase in mitochondrial H_2_O_2_ formation. However, the reduced mitochondrial activity may also decrease ATP synthesis via oxidative phosphorylation and alter bioenergetics of mandibular mitochondria [32]. All of this leads to hypoxic stress and can interfere with the healing process. It cannot be ruled out that mitochondrial alternations as well as nitrosative/oxidative changes observed in our study may be linked not only to titanium, but also to aluminum and vanadium, which are components of the Ti4Al4V alloy. These elements not only improve the biomechanical properties of the titanium implants, but also have pro-oxidative effects [35]. In addition, the observed changes in the redox balance may also be due to increased tissue remodeling and increased protein metabolism. However, fragments of the periosteum-like tissue in the study group were collected 3–5 months after the osteosynthesis and, therefore, neither inflammation nor oxidative stress associated with enhanced tissue remodeling were observed.

Oxidative damage caused by Ti4Al4V occurs, however, not only in proteins, but also in lipids (↑MDA (+204%) in the periosteum-like tissue of study group). MDA is considered a marker of osteoclastic activity [33], which may suggest intensified osteolytic processes around titanium miniplates and screws. Similar results were obtained by Kinov et al. [44] in the study of peri-implantation tissues in patients with aseptic loosening of hip prosthesis. Such changes were explained by high concentration of titanium denture wear products which exacerbate oxidative stress level [44]. This hypothesis is also confirmed by the study of Wang et al. [45] who found that exposure to implant wear products stimulates the production of ROS by macrophages and osteoclasts.

To sum up, our study is the first to observe abnormal mitochondrial activity, disturbances in glutathione metabolism as well as nitrosative stress in patients treated with titanium mandibular fixations. Although exposure to Ti4Al4V titanium alloy induces glutathione defense mechanisms, patients suffer oxidative damage to the proteins and lipids. Titanium implants cause oxidative/nitrosative stress, which may indicate the need for improvement of miniplates and screws used for osteosynthesis. In addition, antioxidant supplementation of patients with titanium facial implants is worth considering and further studies are needed to better understand the phenomenon and indicate the benefit of the use of antioxidant supplements.

Finally, it is important to note certain limitations of our work. We only evaluated the selected nitrosative stress and mitochondrial function markers, so we cannot fully characterize the contribution of free radicals to oxidative damage induced by titanium fixations. Although a carefully selected group of young people (aged 20–28) with similar types of mandible fractures qualified for the study, it cannot be excluded that the observed disturbances of redox homeostasis may be caused, to some extent, by healing of mandibular fractures and may result in intensified protein metabolism.

## Figures and Tables

**Table 1:** The percentage content of carbon (C), oxygen (O), sodium (Na), silicon (Si), phosphorus (P), sulfur (S), chlorine (Cl), aluminum (Al), titanium (Ti), and vanadium (V) on the surface (points 1, 2, and 3) of the periosteum-like tissue in the study group and periosteum in the control group. * study group vs. control, *p* < 0.05; Student’s *t*-test.

Percentage Content on the Surface of the Sample (%)	Control			Study		
	Point 1	Point 2	Point 3	Point 1	Point 2	Point 3
C	50.01 ± 8.31	51.14 ± 9.34	49.98 ± 9.23	47.21 ± 9.36	50.76 ± 2.85	49.56 ± 5.84
O	47.13 ± 6.88	46.62 ± 7.24	48.16 ± 8.06	50.63 ± 9.76	47.17 ± 7.87	47.70 ± 3.11
Na	0.99 ± 0.16	1.23 ± 0.21	0.92 ± 0.18	0.98 ± 0.18	1.22 ± 0.23	0.95 ± 0.16
Si	0.43 ± 0.06	0.24 ± 0.04	0.11 ± 0.02	0.13 ± 0.02	0.12 ± 0.02	0.61 ± 0.11
P	0.20 ± 0.03	0.14 ± 0.02	0.08 ± 0.01	0.17 ± 0.03	0.09 ± 0.01	0.07 ± 0.01
S	0.48 ± 0.09	0.35 ± 0.06	0.27 ± 0.05	0.30 ± 0.05	0.18 ± 0.03	0.47 ± 0.09
Cl	0.30 ± 0.05	0.24 ± 0.03	0.41 ± 0.07	0.25 ± 0.04	0.11 ± 0.02	0.45 ± 0.08
Al	0.00	0.00	0.00	0.24 ± 0.04 *	0.05 ± 0.01 *	0.18 ± 0.05 *
Ti	0.00	0.00	0.00	2.57 ± 0.48 *	1.85 ± 0.34 *	1.06 ± 0.18 *
V	0.00	0.00	0.00	0.14 ± 0.02 *	0.20 ± 0.03 *	0.10 ± 0.05 *
